# State-of-Art on the Recycling of By-Products from Fruits and Vegetables of Mediterranean Countries to Prolong Food Shelf Life

**DOI:** 10.3390/foods11050665

**Published:** 2022-02-24

**Authors:** Sara Nardella, Amalia Conte, Matteo Alessandro Del Nobile

**Affiliations:** Department of Agricultural Sciences, Food and Environment, University of Foggia, Via Napoli 25, 71121 Foggia, Italy; sara_nardella.547821@unifg.it (S.N.); matteo.delnobile@unifg.it (M.A.D.N.)

**Keywords:** fruit and vegetable by-products, food shelf life, sustainable food, by-products recycling

## Abstract

Annually, 1.3 billion tons of food are wasted and this plays a major role in increasing pollution. Food waste increases domestic greenhouse gas emissions mainly due to the gas emissions associated with its production. Fruit and vegetable industrial by-products occur in the form of leaves, peel, seeds, pulp, as well as a mixture of them and represent the most abundant food waste. The disposal of agricultural by-products costs a large amount of money under certain governmental regulations. However, fruit and vegetable by-products are rich in valuable bioactive compounds, thus justifying their use as food fortifier, active food packaging or as food ingredients to preserve food quality over time. The present review collects the most recent utilization carried out at lab-scale on Mediterranean fruit and vegetable by-products as valid components to prolong food shelf life, providing a detailed picture of the state-of-art of literature on the topic. Bibliographic research was conducted by applying many keywords and filters in the last 10 years. Several scientific findings demonstrate that by-products, and in particular their extracts, are effectively capable of prolonging the shelf life of dairy food, fresh-cut produce, meat and fish-based products, oil, wine, paste and bakery products. All of the studies provide clear advances in terms of food sustainability, highlight the potential of by-products as a source of bioactive compounds, and promote a culture in which foods are intended to receive a second useful life. The same final considerations were also included regarding the current situation, which still limits by-products diffusion. In addition, a conclusion on a future perspective for by-products recycling was provided. The most important efforts have to be conducted by research since only a multidisciplinary approach for an advantageous investigation could be an efficient method to promote the scale up of by-products and encourage their adoption at the industrial level.

## 1. Introduction

In later years, the FAO stated that “food loss” comprises a decrease in the quantity or quality of food in the production chain [1]. It typically occurs during the animal and plant production, storage, processing, and distribution stages. In addition, it may be due to an incorrect agricultural practice as well as some phenomena, such as bruising or wilting or inadequate storage [2]. Globally, researchers estimate that, in food supply chains the percentages of food loss in production, postharvest, and consumption stages are 24, 24, and 35%, respectively [3]. “Food waste” is a component of the wider problem of food loss [4] and refers to the decrease in the quantity or quality of food resulting from decisions and actions by retailers, food service providers, and consumers [1]. This is regarding edible food, which is wasted in the second portion of the food supply chain until final consumption. In the UK, for example, the value of wasted products was estimated between USD 1500 to nearly 3000 per ton, depending on whether the waste is generated during processing or final consumption [5]. Food waste causes a large depletion of available land and other environmental concerns, including the unnecessary gas emissions, which are measured by carbon footprint and water wastage [6,7]. Venkat [8] observed that grains, vegetables, and fruits generate 56% of the US waste, but register relatively low emission footprints. 

The current linear model of the economy is based on the concept of the constant supply of products with a short useful life, forcing an increased production to satisfy the consumer’s constant needs. It increases the indiscriminate exploitation of limited natural resources that would yield a significant environmental and economic crisis. In 2009, the FAO organized a forum on “How to Feed the World in 2050”. Based on the prospects of that time period, the world population will increase up to 9.1 billion in 2050, accompanied by an increase of urbanization. This will lead to a rise in annual food production. However, it will only be possible with the implementation of the right investment and agricultural policies [9]. This is only one of many concrete examples, which demonstrate the real entity of the current growth trends. Apart from reducing losses and wastes at all levels of the food chain, it is possible to recycle rather than throw them [10]. Therefore, both economic and environmental impacts may be limited. 

### Recycling of Fruit and Vegetable By-Products

Within the framework of food loss, food industries annually produce tons of by-products during food processing. The most abundant part is represented by fruit and vegetable by-products, which can occur during the pre- and post-harvesting process, preparation, and processing of fruits and vegetables. These industrial by-products are very different from one another due to the difference in industrial processes. For example, grape and olive pomace are derived from wine and oil production, while other fruit by-products are derived from the juice, jelly, and jam industry, for example, from apple, kiwifruit, citrus, passion fruit, mango, etc., as well as from the processing of potato, tomato or carrot. 

By-products have phytochemical compounds with recognized antioxidant and antimicrobial properties [11,12,13,14,15]. Generally, food by-products are used as animal feed or for the production of biomaterials, biofuels, biogas, platform chemicals, and bio-fertilizers [16,17]. However, over time, thanks to the great potential of their active compounds, they have been utilized in several industrial fields (cosmetic, pharmaceutical, and food) [18]. 

**Figure 1:**
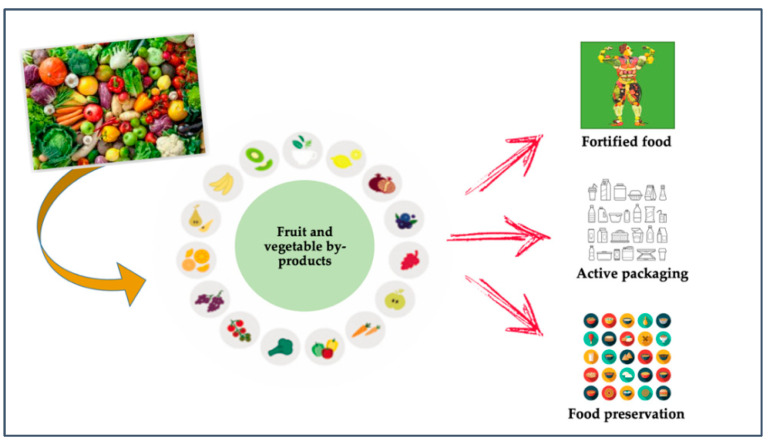
New directions in managing fruit and vegetable by-products.

In regards to the food industry, the goal was to re-introduce the by-products to the production line as raw materials, to obtain new functional products with health benefits or to enhance food preservation or develop active packaging, as presented in Figure 1 [19]. 

The above picture represents the potential applications reported in the scientific literature in the field. In fact, many research studies report the usage of these by-products (i) as ingredients in different types of foods to increase the nutritional value, (ii) as natural preservatives to maintain food quality, (iii) as a source of bioactive agents to develop films and coatings with antimicrobial and/or antioxidant properties. 

Specifically, with regards to the by-products for food preservation, it is interesting to observe that literature data are very abundant. Some of these studies are related to the active packaging systems, which are aimed at prolonging food shelf life. Additionally, other studies are focused on by-products, which are directly applied to food in order to preserve its quality during storage. Here, we provide some examples. Aloui et al. [20] developed a film incorporated with an extract of tomato pomace, while Kanmani and Rhim [21] added grapefruit seed extract into an agar film. Torres-León et al. [22] and Kanatt and Chawla [23] tested the effect of mango by-product-based films on peaches and chicken meat, respectively. Other authors investigated the usage of winemaking by-products on fruits and vegetables [24,25] and on fishery products [26,27]. Madzuki et al. [28] and Gallego et al. [29] tested tomato by-products added with film and coating on the deterioration of calamansi and pork meat, respectively. Moreover, there are various studies on different types of olive milling by-products [30,31]. In regards to by-products that are added to food in order to prolong the shelf life, the numerous applications found highlight that their effects are strictly dependent on the type of by-products and on the food characteristics. Therefore, the present review aims at collecting all of the information available in the literature during the last 10 years, which deal with specific applications of fruit and vegetable by-products to prolong food shelf life. To better organize the work, the studies were divided by the type of food, thus including dairy food, fresh-cut produce, vegetable-based processed food, meat, fish, and cereal-based products. In each paragraph, the reader can find details regarding the typology, concentration, and technique to apply by-products to food. In addition, the main results regarding the effects of by-products on food quality were highlighted. This provides a real map of the most effective by-products and food sectors, where by-products could find the concrete possibility of recycling. The final considerations regarding the current situation and future trends are also reported in the conclusions.

## 2. Materials and Methods

In this review, electronic literature searches were conducted using the online library “opac.unifg.it”, which collects files from several databases including PubMed, Google Scholar, and Science Direct databases. Numerous search terms have been used, including keywords as “fruit by-products and food shelf life”, “vegetable by-products and food shelf life”, “antioxidant activity of food by-products”, and “antimicrobial activity of food by-products”. More specific search terms were also used, as “pomegranate by-products in food products”, “grape pomace and food shelf life” or “fruit by-products and meat shelf life”. The selected time interval was 2010–2022, since the treated subject is quite recent and the aim of the current review is to collect the latest lab-scale trends. The research process uncovered numerous articles, and among them, 86 articles were used for the current overview. Literature data focused on the potential re-utilization of fruit and vegetable by-products, and how these resources can be used as ingredients in food products to prolong food shelf life. The studies cited in this overview are grouped by the type of food, in which by-products are added (dairy food, horticultural food, vegetable food, meat, fish food, and cereal food).

## 3. By-Products and Dairy Food Applications

Dairy products represent a large and differentiated group of food products. The quality of raw material is the first aspect that must be ensured when referring to dairy products. Furthermore, each type of product is associated with specific defects. However, it is possible to identify some factors that are common to a range of goods. In fact, high fat products are prone to oxidation. In addition, dairy products can be subjected to enzymatic, chemical degradation or spoilage by bacteria, molds, and yeasts that grow at a refrigerated temperature [32]. At present, there are many innovative treatments and packaging technologies, which can limit the degradation of dairy products and extend their shelf life. In this regard, fruit and vegetable by-products are a valid option. Only two studies regarding the actual use of fruit and vegetable by-products to prolong the dairy product shelf life are reported in the literature, one study with olive by-products and the other with grape by-products. The main research results are presented in Table 1.

### Olive and Grape By-Products

Olive oil extraction produces a variety of solid and liquid by-products, which can be grouped in olive pomace, olive mill wastewaters, olive leaves, and olive stones, and seeds. In addition to the economic burden for producers, olive oil by-products represent a severe environmental problem for their safe disposal. Simultaneously, these are rich in bioactive molecules as phenolic compounds with antioxidant and antibacterial activity [33], which can be used in the food industry as a source of natural preservatives. Rolia et al. [34] used olive phenolic extract, obtained by liquid–liquid extraction with ethyl acetate, to limit the growth of spoilage bacteria in “Fior di latte” cheese. Phenolic extract was added to the brine of packaged cheese, in two different concentrations, 250 and 500 μg/mL. The maximum microbial load tolerated for *P. fluorescens* was reached at nearly 13 and 15 days of storage for 250 and 500 μg/mL loaded samples, respectively, compared to about 11 days, which was found for the untreated cheese. For Enterobacteriaceae, the threshold limit was reached at nearly 14 and 16 days of storage for 250 and 500 μg/mL added samples, respectively, whereas it was reached at about 10 days for the traditionally packaged cheese. Therefore, it can be asserted that this olive by-product extract can be advantageously used to extend the shelf life of “Fior di latte” from 2 to 4 days, by limiting the main spoilage microorganisms. 

Grape processing by-products are generated from the winemaking process. It typically occurs as pomace consisting of a mixture of residual seeds and skins, which are rich in polyphenols, dietary fibers, citric acid, ascorbic acid, tocopherols, limonoid, and other trace compounds. These mixtures also have a strong antimicrobial activity against both Gram-positive and Gram-negative bacteria, antiseptic, germicidal, fungicidal, and anti-viral properties [35]. For this reason, wine grape pomace can represent an excellent alternative to synthetic compounds in prolonging food shelf life. Tseng and Yanyun [36] examined the feasibility of using wine grape pomace as a source of antioxidant dietary fibers and polyphenols in yogurt for the improvement of its nutritional value and enhancement of its storability. In this study, dried whole grape pomace, pomace liquid extract (LE), and freeze-dried liquid extract (FDE) were investigated. Peroxide value, as an indicator of oxidation, increased during storage and after 3 weeks. In addition, 3% grape pomace added with yogurt showed the lowest oxidation value. The results are interesting, even though it is still necessary to further examine the mechanisms and methods of retention of total phenolic content and radical scavenging activity in the product.

## 4. By-Products Applied to Fresh-Cut Produce

Minimally processed foods represent a great source of vitamins, minerals, dietary fiber, and other constituents. Their peculiarity is that as living entities, their metabolic processes, such as respiration, transpiration, and biochemical transformations continue after the harvest. Therefore, fresh produce quality decreases throughout time, until it becomes unacceptable for consumption. However, these processes can be slowed down by manipulating the process and storage conditions. Due to the advent of increasingly innovative technologies, it is possible to extend their shelf life over and beyond the harvest season [37]. This section is regarding the actual use of fruit and vegetable by-products, which are used as a dipping solution or food ingredient, with the aim of extending the fresh-cut food shelf life. All of the case-studies are presented in Table 2 and reported in detail below.

### Tomoto Papaya, Grapefruit, and Pomegranate By-Products

Tomato by-products are a good source of chlorophylls and phenolic compounds with antioxidant and antimicrobial activities [38]. Martínez-Hernández et al. [39] investigated the effects of lycopene microsphere based dipping solutions on the evolution of the physicochemical, microbial, and bioactive quality of fresh-cut apples during refrigerated storage. Lycopene was obtained by thermal extraction from tomato by-products. The Browning Index showed that the treated samples of lycopene microspheres maintained a lower value until the 9th day of storage. In particular, this is shown in the sample dipped in the solution with the highest concentration of lycopene microspheres when compared to the water dipped control sample and the ascorbic acid dipped sample. Microbial loads of the control sample showed mesophilic, yeast, and mold increments after 9 days, similar to fresh-cut apples dipped in low lycopene microspheres solutions. Whereas, the sample dipped in the highest concentration of lycopene microspheres, showed no microbial increment during the storage period. 

Papaya peel is also used to inhibit the food browning process. Papaya is a tropical fruit, in which its processing by-products mainly consist of peels and seeds. They are rich in bioactive compounds [40]. Faiq and Theerakulkait [41] evaluated the effects of dipping in the papaya peel crude extract and distilled water-based solution on the browning inhibition in potato, banana, and apple slices. Papaya peel extract was obtained by stirring a mixture of papaya peel with distilled water and then centrifuging. The authors reported that during storage, the extract had an higher percentage of browning inhibition in potato slices when compared to banana and apple slices. 

In regards to grapefruit seed extract, Kim et al. [42] conducted a study on its antimicrobial efficacy against *E. coli* O157:H7, *S. typhimurium*, and *L. monocytogenes* inoculated in fresh-cut lettuce, alone and in combination with malic acid. Fresh-cut lettuce was stored at 5 °C, and its microbial quality was monitored over a period of 14 days. Regarding *E. coli* O157:H7, all of the treated samples showed more than 4.4 log CFU/g reductions. Concerning *S. typhimurium*, all of the treatments had more than 4.1 log reductions. Moreover, *L. monocytogenes* was subjected to a significant reduction of its viable cell concentration in all of the treated samples. Furthermore, it was observed that the combined treatment was more effective for the reduction of all the tested foodborne pathogens.

Pomegranate by-products mainly consist of seeds and peels, that originate from the industrial production of juices and jams. They are rich in active compounds, in particular, anthocyanins and hydrolysable tannins, with high antioxidant and antimicrobial activities [43]. Belgacem et al. [44] tested pomegranate peel extract as a natural antimicrobial to reduce the growth of *L. monocytogenes* inoculated on fresh-cut melons, apples, and pears via dipping. Fresh fruits were peeled, cut, and inoculated with the pathogen. Then, the inoculated samples were dipped in solutions at several extract concentrations. Regarding the fresh-cut apples, the sample without the extract showed an increase of the population of *L. monocytogenes* during 7 days of cold storage. On the contrary, the extract efficiently reduced the population of the pathogen. Moreover, the viable cell concentration reduction was reported as higher for samples with the higher concentration of added extract. In regards to fresh-cut pear, except for the sample at the lowest extract concentration, all of the other treated samples showed a significant reduction of *L. monocytogenes* when compared to the control sample. As expected, the sample at the highest concentration was the most effective. Moreover, in fresh-cut melon, after 7 days of storage, the sample at the highest concentration showed the most significant reduction of the tested pathogen. Elsherbiny et al. [45] also investigated the efficacy of a methanol extract of pomegranate peel applied as curative and preventive treatments to control *Fusarium* dry rot on potato tubers, using a dipping in five concentrations of extract solution or distilled water (control). The authors reported that, in curative application, the extract caused a significant reduction of dry rot development of potato tubers when compared to the control. The best reduction was obtained for the sample with the highest extract concentration. Moreover, it significantly reduced the diameter of dry rot lesions in potato tubers in the preventive application when compared to the control. Very recently, other authors tested pomegranate peel as it is, without producing any extract. In this case, Lacivita et al. [46] evaluated the effects of pomegranate peel powder at two concentrations, on the quality decay of a mixture of fresh-cut fruit salad (nectarine and pineapple) in fructose syrup, stored at 4 °C for 4 weeks. Microbial analysis showed that for the first 8 days, all of the samples maintained approximately the initial mesophilic and psychrotrophic bacteria concentration, whereas at the end of the storage period, the control sample had a significantly higher bacterial proliferation. The control salad was microbiologically acceptable for about 24 days, whereas both of the treated samples were below the microbial threshold until the end of storage. Therefore, the lowest concentration of pomegranate by-product is enough to ensure a longer microbial stability of fresh-cut fruit salad when compared to the control. In particular, at the end of the storage period, the treated salad had a significantly lower yeast concentration when compared to the control, especially the sample with the highest concentration of peel. It was also observed that the peel was very effective on lactic acid bacteria, which were lower than the control at the end of the storage. The overall sensory quality of the control sample lasted for 3 weeks, whereas the active samples lasted longer. In conclusion, it can be stated that the pomegranate peel capacity can be considered as a broad-spectrum since it had the capability of exerting a good antimicrobial and antifungal activity. These by-products can represent a great alternative to synthetic compounds, which are generally recognized by the scientific community as effective sanitizers of fresh-cut fruits [44]. 

## 5. By-Products Applied to Vegetable-Based Processed Products

Various types of vegetable-based processed foods, such as wine, olive-based pâté, hazelnut paste, and different refined oils, are presented in this section. Each of these foods has a peculiar mechanism of deterioration and specific shelf life since chemical, microbiological, and physical changes can occur during storage. In recent times, many studies have been conducted on the feasibility of preserving these different food products with natural compounds derived from by-products. Table 3 briefly presents the case-studies that are detailed in the subsequent two paragraphs.

### 5.1. Olive By-Products

Sulfur dioxide (SO_2_) is commonly added to commercial wines at the time of bottling, in order to confer microbiological and oxidative stabilities [47]. Ruiz-Moreno et al. [48] investigated the antioxidant and antimicrobial activities of hydroxytyrosol-enriched extract obtained from olive mill waste, as a potential alternative to SO_2_ in winemaking. The authors concluded that the hydroxytyrosol-enriched extract itself is not sufficient for the replacement of SO_2_ in wines, but a combination of SO_2_ and hydroxytyrosol-enriched extract could be a valid solution. In fact, the analyses showed that *Saccharomyces cerevisiae* delayed its growth only with SO_2_. *Hanseniaspora uvarum* and *Pediococcus damnosus* were completely inhibited by SO_2_, whereas the extract only delayed their growth. The extract resulted in inefficiency against *Lactobacillus plantarum*. 

Bouaziz et al. [49] conducted a study on different types of olive leaf extracts (enzymatic hydrolysate leaf extract (EHE), acetylated hydrolysate extract (AHE), and pure oleuropein), in refined olive oil (ROo) and refined olive-pomace oil (RPo) during storage, then compared them to extracts with α-tocopherol. Both of the oils that were added with EHE showed the lowest oxidation value. The rise in oxidation of the control was similar to the AHE added with oil. Finally, the oxidative resistance improved with the addition of olive leaf phenolic extracts, especially for the EHE loaded sample. Furthermore, after 6 months of storage, the oxidative resistance was lower for the two refined oils added with oleuropein, whereas it fell to zero in the control sample, as well the extracts enriched with α-tocopherol and AHE. Therefore, it can be stated that enrichment with olive leaf extract reduced oil rancidity. 

Other authors [50] evaluated the effect of olive leaf extract on non-thermally stabilized olive-based pâté during storage, as compared to the butylated hydroxytoluene (BHT). In regards to the microbial analysis, the absence of pathogens (*Clostridium* and *Listeria* spp.) and contaminants (*Pseudomonas* spp. and Enterobacteriaceae) was observed. As the samples of olive-based pâté were not subjected to thermal stabilization, cultivable bacteria, yeasts, and molds were detected during sample production and storage. However, their growth was affected by the addition of the extract and refrigeration storage. The main microbial groups registered a significant reduction of the viable cell concentration in samples added with the extract, especially with 1.0 g/kg. Therefore, the authors reported that the samples were suitable for consumption, from a microbiological point of view, during refrigeration storage for 120 days under modified packaging conditions, compared to the 90 days of the untreated or BHT-added samples.

### 5.2. Potato, Grape, and Pomegranate By-Products

Potato by-products usually occur, among other residues, as peels, which are derived from many industrial potato processes. Potato peel is a rich source of bioactive compounds due to its high content in phenolic compounds with recognized health-promoting properties [51,52]. Samotyja [53] evaluated the antioxidant properties of two different cultivars (Jazzy and Gala) of potato peel extracts on rapeseed and sunflower oil. In regards to rapeseed oil, potato peel extracts of both cultivars inhibited the formation of hydroperoxides, showing higher efficiency when compared to BHT and butylated hydroxyanisole (BHA). Among the tested extracts, 80% ethanol extract was the most efficient. The extracts also delayed the formation of conjugated diene hydroperoxides in oil. In regards to sunflower oil, ethanolic extracts showed a moderate activity against hydroperoxides formation. Moreover, by increasing the extract concentrations, the activity against hydroperoxides formation increased. All of the investigated extracts were capable of delay conjugated diene hydroperoxide formation. Furthermore, all of the extracts significantly reduced hexanal formation when compared to the control. Therefore, potato peel extracts, are not only capable of delaying primary oxidative changes, but also have a positive effect on retarding oil rancidity. 

In regards to grape by-products, Spigno et al. [54] investigated the feasibility of using a grape marc phenolic freeze-dried extract to protect commercial hazelnut paste against oxidation, crude, and encapsulation into different nano-emulsion-based delivery systems. The authors asserted that phenolic grape marc can inhibit hazelnut paste oxidation and consequently improve its shelf life, even though the antioxidant effect was limited since the paste split in two phases after 5 weeks and the extract was separated. The extract did not solubilize completely in the dark fluid hazelnut paste added with emulsifiers. The encapsulation process reduced the antioxidant activity of almost all of the extracts. All of the tested formulations were no longer active after 83 days. The oil in water nano-emulsion resulted in the best encapsulation solution due to its efficiency. 

Pomegranate peel extract exerted a significant role in the preservation of seed oil. This is the result of the case study by Drinić et al. [55]. The authors investigated the effect of pomegranate peel extract, alone and combined with BHT, on the antioxidant stability of pomegranate seed oil. At the end of storage, oil with the combination of antioxidants (0.05% pomegranate peel extract and 0.01% BHT) had a significant lower oxidation when compared to the pomegranate peel extract added with oil. Thiobarbituric acid reactive substances (TBARS) showed that at the end of storage, oil added with pomegranate peel extract showed the highest percentage of oxidation inhibition, followed by the combination of antioxidants and BHT. 

## 6. By-Products and Meat Applications

The shelf life of meat products is influenced by many factors, which are complex and interconnected. Microbial growth is one of them, and the types of microorganisms that can be found in meat depend, among other things, on animal species, personnel sanitation, type of packaging, storage time, and temperature [56]. Lipid oxidation is the major cause of chemical alteration during storage. It causes irreversible changes in taste, flavor, color, and texture of the products, resulting in a shelf life reduction. Moreover, protein oxidation causes the loss of sensory properties, essential amino acids, and protein digestibility. Modified atmosphere packaging and synthetic antioxidant and antimicrobial substances are widely used in the food industry to improve the meat product shelf life. Nevertheless, synthetic compounds are known as potentially toxic. Therefore, natural extracts can represent a good alternative to protect these foods.

### 6.1. Olive Mill Wastewater, Pomace, and Seed Extract 

As previously reported, tomato pomace has shown good bioactive properties. Olive pomace is an important natural source of phenolic compounds. Pomegranate seeds are known to have powerful antioxidant compounds. Finally, grape pomace is very rich in antioxidant and antibacterial agents. All of these by-products were adopted, mainly in the form of extracts, with slight effects on the quality of meat-based food. In general, all of them, and in particular grape by-products, are capable of preserving meat products against lipid oxidation, protein oxidation, and microbial growth (Table 4). Nevertheless, in some cases, it is better to combine them with other preserving methods to increase their effects.

In the study by Andrés et al. [57], the authors evaluated the shelf life of lamb meat patties added with aqueous extracts from tomato, olive, pomegranate, and grape by-products stored in retail sale conditions. The authors reported that free thiols consistently decreased after 7 days of storage, even though the extract-loaded samples showed the highest values at the end of the observation period. Counts of mesophilic and psychrotrophic bacteria in lamb patties with extracts were significantly lower than the control and sodium ascorbate added samples, even though the antimicrobial effect of the extracts was less evident for Enterobacteriaceae and lactic acid bacteria. In a second study, Andres et al. [58] evaluated the in vitro antioxidant potential of tomato pomace extracts and then the effect on the shelf life of lamb meat packaged under modified packaging conditions (MAP). After 7 days of storage, the TBARS values of meat treated with the extract obtained using ethyl acetate and with the extract obtained using ethanol significantly increased, with no significant differences from the control. Free thiols significantly decreased during storage. Moreover, microbiological analysis showed that the aerobic count in lamb meat remained under the microbiological limits in fresh meat, for both mesophilic and psychrophilic bacteria. The final values of lactic acid bacteria were also substantially lower than the imposed limit. 

Better results were found by Selani et al. [59], who investigated the effects of Isabel and Niágara grape seed and peel extracts on lipid oxidation of raw and cooked chicken meat vacuum-packed and stored at −18 °C for 9 months. The authors reported that both natural extracts were similar to commercial antioxidants in preventing lipid oxidation in raw and cooked chicken meat and their effects were more evident in cooked samples. According to Selani et al. [59], other authors also assessed the effects of grape seed extracts. Kulkarni et al. [60] compared three levels of grape seed extract as a commonly used antioxidant, in pre-cooked, frozen, and stored beef and pork sausage. Based on sensory characteristics, instrumental color, and TBARS values, the authors concluded that the sample added with 100 and 300 ppm of extract was generally as good as propyl gallate in maintaining product quality during the 4 months of storage. 

Lorenzo et al. [61] evaluated the effect of grape seed and chestnut extracts and BHT on physico-chemical and microbiological changes, as well as lipid oxidation during the ripening process of dry-cured sausages. To this aim, the extracts were mixed with meat during the initial phases of processing. The authors asserted that the grape seed extract was the most effective antioxidant of dry-fermented sausages. In fact, they reported that the TBARS values decreased when compared to the control. However, compared to the control and chestnut extract-treated sausages, grape seed extract improved lipid stability. Total viable count and lactic acid bacteria increased during the first 19 days of ripening and remained stable until the end of the curing process. The highest lactic acid bacterial counts were observed in sausages of the chestnut extract group and control batch. At the end of the curing process, mold and yeast counts were higher in control and grape seed extract samples than in BHT and chestnut extract-treated sausages.

Grape pomace extract was found less effective than seed and peel extracts by Garrido et al. [62]. These authors evaluated the effects of two types of grape pomace extracts on the physico-chemical characteristics of pork burgers and their preservative capacity. They reported that total viable count, psychrophilic bacteria, and total coliform count were not affected by the extract addition. In fact, the samples were microbiologically unacceptable after 6 days. Nevertheless, compared to the extract obtained using methanolic extraction, the extracts obtained using the high–low instantaneous pressure and high–low instantaneous pressure + methanolic extraction showed a stronger antioxidant effect when added to meat. 

Garcia-Lomillo et al. [63] evaluated the effect of milled red and white grape skin wine pomaces on beef patties, which are stored for 15 days in high oxygen modified atmosphere against protein oxidation, in comparison to the protective effect of sulfites. The analysis on beef proteins showed that patties added with sulfites had the lowest accumulation of protein radicals. The addition of white grape skin wine pomace to the beef patties resulted in higher radical intensity, whereas the addition of red pomace caused no effect compared to the sample added with water (control). Moreover, while the radical intensity of control and white pomace added with patties increased during storage, those added with sulfites or red pomace were constant. The authors concluded that red pomace effectively protected against protein oxidation. 

In regards to olive mill wastewater, Chaves-López et al. [64] evaluated its effect against the undesired household fungi that may grow on the surface of Italian dry fermented sausages during ripening, considering its impact on the microbiological and physico-chemical characteristics. The authors reported that all of the microbial groups grew during the observation time, except for Enterobacteriaceae, which decreased. The treated samples showed an extra reduction of fungal growth, which was proportional to the extract concentration. In the enriched samples, only *Penicillium nalgiovense* and *Penicillium chrysogenum* were isolated. Micrococcaceae, yeasts, and molds decreased in the treated batches. However, compared to the control, their proteolysis index was slightly higher. In the presence of olive mill wastewater polyphenols, the volatile compounds derived from microbial esterification and lipid oxidation decreased. Furthermore, TBARS of fortified samples showed reduced values when compared to the control batch. Therefore, the authors found that the surface treatment of fermented sausages with 2.5% by-products addition was effective against some undesired fungal species. 

### 6.2. Olive Leaf Extracts

Olive leaf extract was also greatly used at lab-scale to prolong the shelf life of several meat-based foods, which are considered a valid alternative to synthetic additives. The extracts are capable of maintaining both chemical and microbiological safety. Therefore, they can enhance meat quality in the same way or even better than synthetic compounds. In many cases, it was reported that their positive effect was more evident by increasing their concentrations (Table 4).

Specifically, Baker [65] conducted a study to establish the optimum concentration of olive leaf extract in minimizing the oxidative and microbiological deterioration of Karadi lamb patties. The author reported that compared to the untreated sample, the treated samples with 1, 2, 3% of extract had significantly lower TBARS values. The bacterial counts increased during storage. However, for the treated patties, microbial proliferations were significantly lower when compared to the control sample, until the end of the tested period. The authors found that 1% of treated patties also showed the best overall acceptability. In the same context, Djenane et al. [66] used Algerian wild olive leaf extracts as an enrichment of Halal minced beef at two different levels (1 and 5%, *v*/*w*) and evaluated their effects on microbiological safety during 6 days of retail. The authors reported that the addition of 5% extract showed the strongest antimicrobial activity. In addition, at the end of the storage period, the microbial count found in the treated samples was still not near the critical microbiological threshold. The TBARS values of all the tested samples also decreased by increasing the extract concentration. Other authors recently [67] assessed the effects of olive leaf extracts on physico-chemical properties and microbial quality of chilled poultry meat. To this aim, meat was dipped for 15 min in the extract at concentrations of 0.25%, 0.5%, and 1%. Herein, it can be asserted that olive leaf extract has the capability of maintaining the chemical and microbiological quality of chilled poultry meat. In fact, compared to the control (meat without the extract), the total volatile basic nitrogen (TVBN mg/100 g) in poultry meat after the treatment was significantly reduced, especially in 1% of the treated sample. Moreover, this last sample reached the unfit limit for TVN after 15 days over 6 days for the control. The TBARS values of all the samples increased during storage. However, compared to the control samples, the treated product had noticeably lower TBARS values. The extract also decreased the total aerobic plate count. Compared to the lower concentrations, 1% was more effective. Moreover, the extract, especially 1%, positively delayed the growth of psychrophilic, Enterobacteriaceae, Staphylococcal, as well as the mold and yeast counts. Elama et al. [68] investigated the effects of oleuropein from olive leaf extract on lipid peroxidation in frozen bovine hamburger, compared with sodium erythorbate. Oleuropein was mixed at different concentrations with meat during the hamburger preparation. The authors reported that the amount of oxidation products increased for both control and treated samples (with 0.25, 0.5, and 0.75%) during 6 months of storage. However, at the end of storage, the amount of oxidation products was found as lower in the treated samples than in the control. The sample which showed the best results was the one with 0.5% oleuropein. 

The effects of destoned olive cake on the physico-chemical, microbiological, and sensory quality of beef patties during cold storage were also assessed by other authors [69]. Samples were prepared by mixing minced beef with olive by-products at several concentrations. Compared to the untreated sample, the DPPH radical scavenging activity values after 14 days of storage of fortified patties at a concentration above 2% were found as higher. The TBARS values of all the samples increased during the storage period. However, compared to the untreated samples, the TBARS values observed for the treated beef patties were lower. Moreover, it was found that the by-products effect is concentration dependent. Furthermore, the authors observed that all of the fortified samples had a significantly lower total plate count than the control sample. Therefore, the authors reported that the incorporation of destoned olive cake in beef patties could prolong their shelf life up to 14 days. To conclude the state-of-art of olive by-products, the case study by Nieto et al. [70] can be also cited. These authors evaluated the effects of different hydroxytyrosol extracts on chemical and sensory properties of low-fat frankfurter chicken sausages during 21 days of storage at 4 °C in a modified atmosphere. The chicken sausage formulation includes olive oil as a fat substitute, walnut as a macronutrient, and hydroxytyrosol extract as an antioxidant. The extracts were added to the homogenized meat, then mixed. The authors reported that the TBARS values of all the samples increased during the storage period. However, compared to the control sample, the oxidation was significantly lower in extracts added with sausages. Compared to the control with pork fat, control with walnut, and control with walnut and olive oil, the extracts significantly reduced the thiol concentration since the start of the storage period. Therefore, to conclude, the addition of walnuts and extracts was useful for the prevention of lipid and protein oxidation of sausages, especially when olive oil was used rather than pork fat. 

## 7. By-Products Applied to Fish-Based Products

Fish food represents a highly healthy food, as they are an abundant source of high biological value proteins, long chain polyunsaturated fatty acids, and other nutritional components. In fact, color and lipid oxidation are the main factors for quality deterioration in fishery products during storage. The pH values in fish, which do not decrease as in meat products, cause enzymatic systems to be highly active, thus increasing their vulnerability to bacteria [71]. The shelf life of fish products is usually extended with refrigeration, freezing or thermal inactivation. Synthetic additives are frequently used to enhance their shelf life, although nowadays, food industries are searching for natural alternatives and have found them in by-products (Table 5).

### 7.1. Peel, Seed, and Leaf Extracts

Viji et al. [72] investigated and compared the effects of citrus peel extract and mint extracts to prolong the shelf life of washed, beheaded, and eviscerated Indian mackerel during chilled storage. The authors reported that TVBN increased significantly in all of the samples during storage. The rate of lipid hydrolysis was substantially lower in treated fish, whereas the level of free fatty acids and peroxide values in the sample remained slightly lower than the control sample. The TBARS values of all the samples increased gradually during storage. However, at the end of the observation period, the TBARS value of the sample with mint extract was the lowest one. Moreover, the extracts were capable of slowing down bacterial growth. Therefore, the treatments extended the shelf life of refrigerated mackerel by 2 and 5 days with citrus and mint extracts, respectively. 

Özen and Soyer [73] evaluated the efficiency of green tea extract, grape seed extract, and pomegranate rind extract in limiting lipid and protein oxidation in minced mackerel, during 6 months of frozen storage. Peroxide values increased for minced mackerel added with the extracts and the control until the 4th month of storage, then declined rapidly. Among the extracts, pomegranate was the most effective. TBARS of the BHT treated sample showed only a slight increase, followed by the sample with pomegranate extract. However, at the end of frozen storage, compared to the control, all of the samples loaded with antioxidants showed significantly lower TBARS values. Compared to the control, the carbonyl content of all the antioxidant-loaded samples was significantly lower at the end of the 6th month. Furthermore, the changes in total protein solubility showed a progressive decrease throughout the storage period and compared to the control, the antioxidant treated samples showed a significantly higher total protein solubility. The authors concluded that these natural antioxidants improved the oxidative stability of fish and pomegranate extract was the most effective in protecting its quality. Prior to the Özen and Soyer study [73], Özen et al. [74] investigated the effects of natural extracts from by-products on lipid oxidation of fish during 3 months of frozen storage. The authors used grape and pomegranate seed extracts added to minced chub mackerel muscle. In addition, they reported that peroxide values increased for all of the tested samples during the storage period, especially for the pomegranate loaded sample, whereas no significant increase was observed for the sample with grape seed extract. Moreover, compared to the antioxidant treated samples, the TBARS values of the control fish were significantly higher at the end of the tested period. 

Hasani and Alizadeh [75] evaluated the effects of red grape pomace extract, added at two different percentages (2 and 4%), on quality changes in silver carp fillets during refrigerated storage. The TBARS values of all the samples increased during storage. However, compared to the control, the TBARS values of grape pomace extract-loaded sample were significantly lower, more evidently with 4% of extract. 

Yerlikaya et al. [76] produced an ice with different citrus peel extracts and evaluated their effects on the shelf life and quality of common pandora during refrigerated storage. The authors reported that the TVBN concentrations in the fish stored in ice increased during storage in all of the samples. At the end of the storage, the grapefruit flavedo treatment had the highest TVBN value, whereas the bitter orange flavedo treatment showed the lowest value. Moreover, the TBARS values of samples treated with citrus extracts remained low. The count of total psycrophilic bacteria of all the samples significantly increased during storage, but the bitter orange extract-loaded samples showed the lowest value at the end of the storage. 

No significant results were found by Ali et al. [77], who evaluated the supplementation of extracts from cabbage leaves and banana peels in fish-based products to prevent the formation of potential oxidized free fatty acid and peroxide compounds. Natural extracts were loaded at different concentrations (0.5, 1, and 1.5%) to fish meat balls and then stored both at 4 and −18 °C for 9 days and 2 months, respectively. All of the treated samples showed an increase of peroxide value and free fatty acid values, more evidently when stored at 4 °C. Contrary to Ali et al. [77], Abdel-Wahab et al. [78] investigated with success the antioxidant and antimicrobial properties of clove flower buds, sage leaves, kiwifruit peels aqueous extracts, as well as their mixture, on tuna fish fingers. The extracts mixed at concentrations of 0.1, 0.25, and 0.5% were mixed during processing. The authors observed that the TBARS values significantly increased in all of the samples during 30 days of storage. However, compared to the control sample, the treated fish reached significantly lower TBARS values. Moreover, compared to the 0.25 and 0.5% mixture of the added samples, the carbonyl content increased significantly in the other samples. TVBN values increased in all of the samples during storage. However, compared to the control, the extract mixture of the treated samples showed lower final values. Furthermore, the total bacterial count increased significantly in all of the samples during storage, less than two orders of magnitude in the mixtures of the treated samples. The control reached the threshold limit at day 3, and the BHT-treated fish at day 6. On the contrary, the 0.5% extract mixture of the treated sample reached the threshold limit at day 24, thus demonstrating the great effects of the extracts on product shelf life. 

Miraglia et al. [79] asserted that the extract derived from olive mill wastewater was capable of delaying lipid oxidation, microbial growth, and TVBN on pink shrimp stored at 2 °C. The most remarkable reduction of microbial growth rate in the treated sample was observed for psychrotrophic bacteria.

### 7.2. Peel Powders 

To date, a small number of examples are reported in the literature on the use of peel without any preliminary extraction, before direct application to fish products (Table 5). In this context, two articles can be cited by Panza et al. [80,81]. In the first article, the authors [80] developed a ready-to-cook cod stick breaded with different concentrations of dry olive paste powder and monitored the quality parameters during 15 days of refrigerated storage. The authors reported that compared to the raw and cooked breaded cod sticks without olive paste, the raw and cooked active samples showed an higher antioxidant activity. The microbial cell concentration increased during the storage, in both active and control fish. However, in the control sample, it was significantly higher than in the active samples. The control samples remained slightly lower than the active sample in the overall quality score. When the authors compared both microbiological and sensory limits to determine the product shelf life, they found that the shelf life of active samples was about 12 days and it was longer by 3 days than the control fish. At a later date, Panza et al. [81] evaluated the effects of pomegranate peel powder on the same breaded cod sticks stored at refrigerated conditions. During the 17 days of storage, total mesophilic bacteria of the investigated breaded cod sticks, similar to the psychrotrophic count, gradually increased and reached the unacceptable limit after about 6 days of storage in the sample without the addition of the active powder. At the end of the storage period, the sticks breaded with pomegranate peel powder showed the lowest total bacterial count. The analysis on *Pseudomonas* spp. growth showed that the treated samples never reached the microbial acceptability limit during the storage period, whereas the control sample was already unacceptable after 9 days. Compared to the control, *Shewanella putrefaciens, Photobacterium phosphoreum,* and Enterobacteriaceae growth were limited in all of the treated samples. Therefore, the use of pomegranate peel powder also promoted a significant improvement of microbial stability of cod sticks.

## 8. By-Products and Cereal Food Applications

Cereals are a huge group of food products, among which bakery one represents the main portion. Its distinguished feature is that the recipes contain a significant proportion of wheat flour. Causes of the deterioration of bakery products are microbial spoilage or physical changes, which of course, also cause changes in their sensory properties, thus limiting the shelf life. Among the physical changes, loss or absorption of moisture from the atmosphere, which also causes crumb firming through starch retrogradation, are the most likely to occur during their storage. For this reason, a key function of the packaging of baked products is to control moisture transfer to and from the product [82]. Synthetic additives and modified atmosphere packaging are also widely used to prolong their shelf life. Recent trends include the use of natural compounds obtained from industrial by-products, which are safer and can add value to food products. The three recent case studies are presented in Table 6. 

### Peel, Pomace, and Leaf Extracts

Ismail et al. [83] evaluated the potential of pomegranate extracts and its residues in cookies as a natural preservative and promising food fortifier. The authors reported that higher free radical scavenging properties were observed for extract-loaded cookies when compared to the ones added with residues. Some fortified cookies showed a significant reduction of the microbiological load during the tested period. In addition, samples with extract-supplemented cookies showed a better inhibition rate to lipid oxidation. 

Mehta et al. [84] produced bread and muffins by adding tomato pomace to investigate its effect on their nutritional properties and storage stability. The authors reported that the antioxidant activity of tomato pomace incorporated bread and muffin was higher when compared to their controls. In fact, tomato pomace added with bread showed a shelf life of 5 and 4 days at 10 and 25 °C, respectively, which was longer when compared to the control. 

Difonzo et al. [85] evaluated the effects of olive leaf extract mixed in baked snacks, during both accelerated oxidation conditions and storage. Two different quality levels of oil were used for baked snacks preparation, EVO1 and EVO2. Results of the oxidative stability evaluation showed that the induction period of both oil-added baked snacks was higher when compared to the control. Snacks with EVO1 had a higher activity of hydrophilic fraction than the samples containing EVO2. Moreover, the authors reported that EVO1 showed a higher quality level than EVO2, as the amount of volatile compounds derived from oxidation was strongly lower in the EVO1 added snack. 

## 9. Conclusions

The awareness of human, economic, and environmental impacts caused by food loss and waste encouraged researchers to investigate the feasibility of using industrial by-products to prolong food shelf life. Many compounds present in fruit and vegetable by-products have proven effective in prolonging food shelf life. In particular, the studies collected in this review demonstrated that by-products can be used for inhibiting oxidation processes, microbial growth, as well as physic degradation of food, without compromising sensory properties. Some critical considerations can be highlighted regarding the current situation and future trends.

**Current situation.** At present, it is still difficult to find these kinds of innovative products in the market. This represents a huge lack in the modern world, which is continuously aiming at sustainability. Several causes may lead to this issue. Considering this issue, presumably one of the main reasons is the high-risk investment associated with the recycling of by-products. The industrial implementation for recovery processes is complex and requires a careful evaluation. Currently, it seems as a significantly expensive investment, especially for small- and medium-sized enterprises, as the consumer is not inclined to pay the additional cost. Generally, people tend to assume an incoherent behavior on the matter. In fact, they demand an increased production at the lowest possible cost, and consider it even better when disposable. At the same time, they are increasingly interested in environmental and health matters. This is the reason that nowadays people demand natural preserving additives as a replacement of synthetic ones. However, they commonly tend to identify food by-products with something that is not safe or suitable for human consumption, which is at the end of its life cycle and cannot be reintegrated in the food chain. To date, most of the consumers would be discouraged from buying food with by-products.

**Future trends.** In a novel bio-economy perspective, the promotion of pathways that encourage the recovery of compounds, which are still presenting an added value that otherwise would be lost, is a priority. It is fundamental to dispel the misconceptions, which lead to identifying industrial by-products as trash. As in many other fields, information is the basis of concrete progress. In this perspective, a very important role is played by a holistic research approach, which is capable of identifying the advantages of by-products and their real efficacy. Research needs to simultaneously focus the attention on main interdisciplinary factors that could make industrial food by-products an effective entry point to mitigate the greater food waste problem. The recycling of by-products needs to be approved by the current legislation, and the prospect as new ingredients will depend on new safety and regulatory assessments. In addition, social, environmental, cultural, and psychological influences on consumers’ food choices need to be considered. In particular, consumers should be increasingly aware of the great potential of by-products as a valid support in extending the food shelf life. In this regard, consumers will most likely be willing to pay for the additional cost. 

## Figures and Tables

**Table 1:** Applications of by-products to dairy food.

By-Products	Food	Note	Reference
Olive phenolic extract	Fior di latte cheese	Enhancement of microbial quality and sensory stability.	[34]
Grape pomace extract	Yogurt	Lipid oxidation control, however, the radical scavenging activity decreased during storage.	[36]

**Table 2:** Applications of by-products to fresh-cut produce.

By-Products	Food	Note	Reference
Tomato lycopene	Fresh-cut apples	Reduced enzymatic browning and microbial growth.	[39]
Papaya peel extract	Potato, banana, and apple slices	Browning inhibition in potato slices when compared to banana and apples.	[41]
Grapefruit seed extract	Fresh-cut lettuce	Significant microbial reduction of food pathogens.	[42]
Pomegranate peel extract Peel powder	Fresh-cut apples Potato tubers Fresh-cut salad	It was very effective in reducing *L. monocytogenes* population. It reduced the diameter of dry rot lesions in potato tubers. Microbial control and sensory quality preservation.	[44] [45] [46]

**Table 3:** Applications of by-products to vegetable-based processed food.

By-Products	Food	Note	Reference
Olive (Hydroxytyrosol-enriched extract)	Wine	The extract alone is not sufficient to replace SO_2_, but it was effective in delaying microbial growth.	[48]
Olive leaf extract	Refined olive oils	Oil rancidity reduction.	[49]
Olive leaf extract	Olive-based pâté	The extract enhanced oil oxidative stability.	[50]
Potato peel extract	Rapeseed and sunflower oil	Delay of primary oxidative changes in oil and positive effects on retarding oxidative rancidity.	[53]
Grape marc extract	Hazelnut paste	Capable of inhibiting lipid oxidation.	[54]
Pomegranate peel extract	Pomegranate seed oil	Capable of inhibiting lipid oxidation, above all when combined with other antioxidant compounds.	[55]

**Table 4:** Applications of by-products to meat food.

By-Products	Food	Note	References
Tomato, Olive, Pomegranate and Grape Extract	Lamb patties	Capable of controlling the growth of mesophilic and psychrotrophic bacteria.	[57]
Tomato pomace extract	Lamb meat	Capable of controlling both microbial growth and TBARS values.	[58]
GRAPE Seed and peel extract Seed extract Seed extract and chestnut extract Pomace extract Wine pomaces	Chicken Pork sausage Dry-cured sausages Pork burgers Beef patties	Capable of delaying lipid oxidation without affecting color. Capable of maintaining product quality during the 4 months of storage. Capable of maintaining lipid stability and microbial quality. Antioxidant effects on meat quality. Effective against protein oxidation.	[59] [60] [61] [62] [63]
Olive mill wastewater Leaf extracts Hydroxytyrosol extracts	Italian dry fermented sausages Karadi lamb patties Halal minced beef Chilled poultry meat Frozen hamburger Minced beef Low-fat frankfurter chicken sausages	Reduction of undesired fungal growth on the surface, oxidation control. Effective against oxidation and microbial growth. Effective against oxidation and microbial growth. Effective against oxidation and microbial growth. Effective against oxidation. Significant shelf life prolongation. Capable of preventing lipid and protein oxidation of sausages, especially when olive oil was used rather than pork fat.	[64] [65] [66] [67] [68] [69] [70]

**Table 5:** Applications of by-products to fish food.

By-Products	Food	Note	Reference
Citrus and mint peel extract	Indian mackerel	Considerable effects on fish chemical and microbial quality during storage.	[72]
Pomegranate rind, green tea, and grape seed extract	Minced mackerel	Oxidative stability of fish. Pomegranate extract was the most effective in protecting its quality.	[73]
Grape and pomegranate extract	Fish	Effective against oxidation.	[74]
Grape pomace	Silver carp fillets	Effective against oxidation.	[75]
Citrus peel extract	Ice to store pandora	Effective against oxidation.	[76]
Cabbage and banana peel and leaves	Fish-based products	Partial effects on lipid oxidation.	[77]
Clove flower buds, sage leaves, kiwifruit Peel extracts	Tuna fish fingers	The mixture of antioxidants is effective against oxidation and microbial growth.	[78]
Olive mill wastewater	Rose shrimp	Capable of delaying lipid oxidation, microbial growth, and volatile nitrogen compound formation.	[79]
Olive dry olive paste powder	Breaded cod sticks	Effective in reducing microbial quality decay.	[80]
Pomegranate peel powder	Breaded cod sticks	Very effective in reducing microbial quality decay.	[81]

**Table 6:** Applications of by-products to cereal-based food.

By-Products	Food	Note	Reference
Pomegranate extract	Cookies	Reduction of microbial load and inhibition of lipid oxidation.	[83]
Tomato pomace	Muffins	Control of microbial spoilage and good antioxidant activity.	[84]
Olive leaf extract	Baked snacks	Capable of inhibiting lipid oxidation of oil in baked snacks.	[85]

## Data Availability

Data are available on request.
